# Supplementary material for: A membrane-bound nuclease directly cleaves phage DNA during genome injection
Source: Nature. 2026 Feb 25;653(8115):861–9. doi: 10.1038/s41586-026-10207-1 (PMC13190303; doi:10.1038/s41586-026-10207-1)
Supplement: Supplementary file 1 — This file contains Supplementary Figure 1, Supplementary Tables 1–3 and Supplementary References [file 41586_2026_10207_MOESM1_ESM.pdf]

nature portfolio

https://doi.org/10.1038/s41586-026-10207-1

Supplementary information

A membrane-bound nuclease directly cleaves phage DNA during genome injection

In the format provided by the authors and unedited

Supplementary Figure 1: raw gels

a. Extended Data Fig. 2b, 2f, 4c

anti-GFP

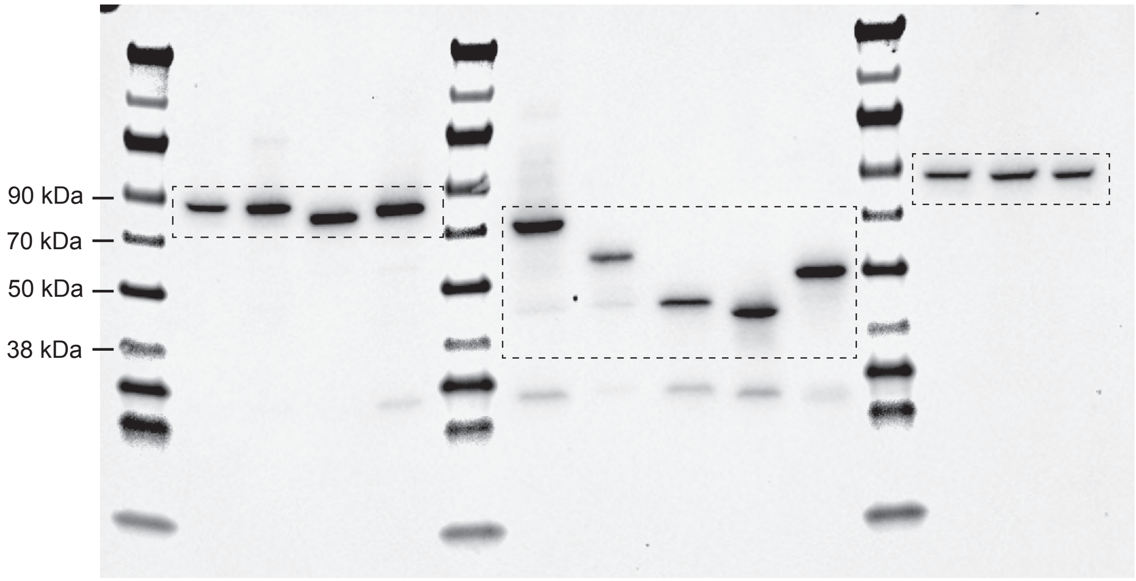

Extended Data Fig. 2b Extended Data Fig. 2f Extended Data Fig. 4c

Coomassie stain for loading control

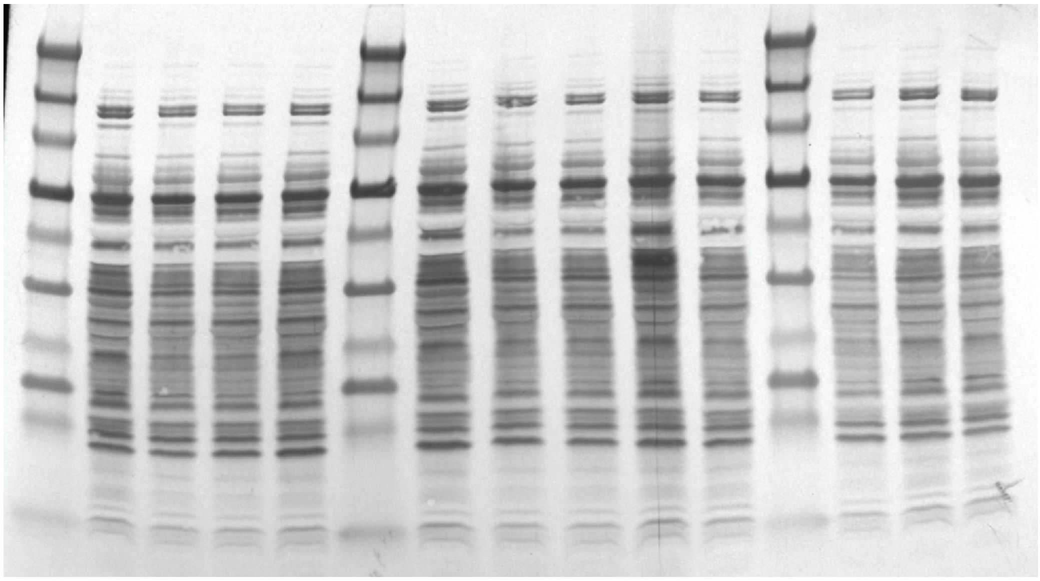

Supplementary Figure 1: raw gels

b. Extended Data Fig. 2c

anti-DnaK

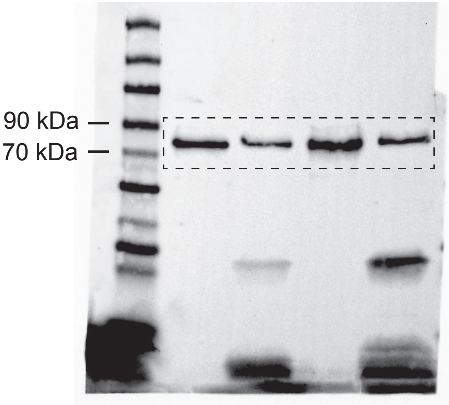

anti-OmpC

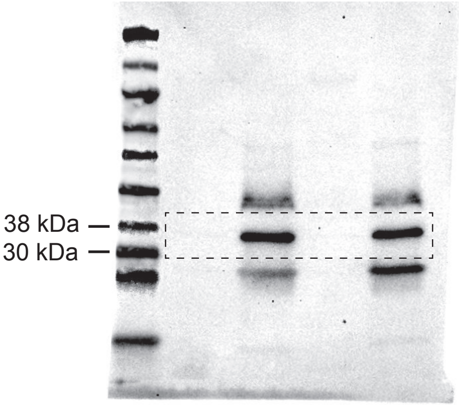

anti-GFP

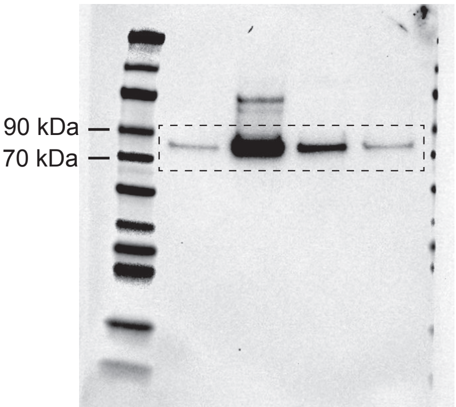

Coomassie stain for loading control

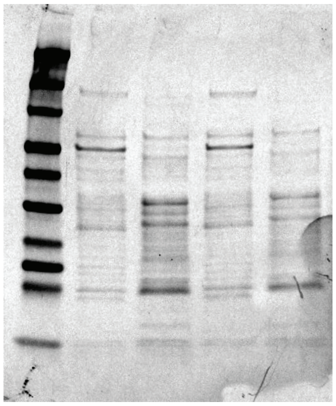

c. Fig. 2d, Extended Data Fig. 3c

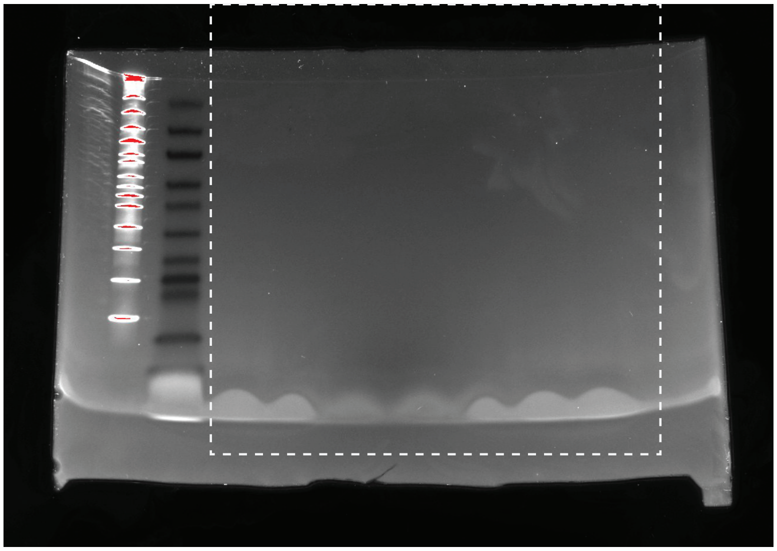

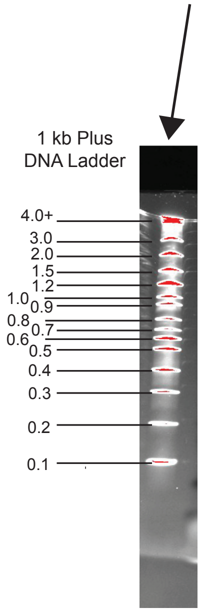

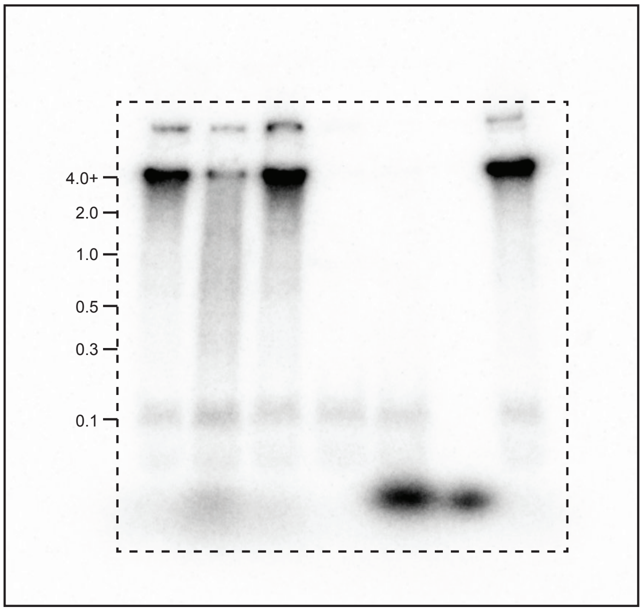

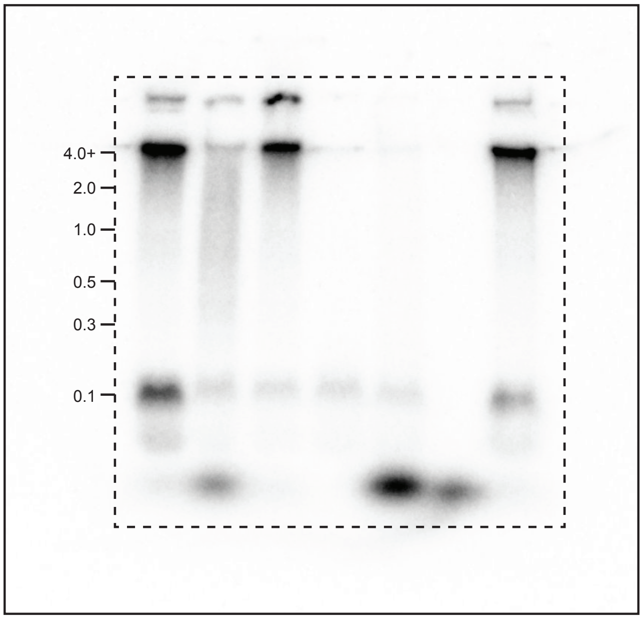

Supplementary Table 1

Phage	Gene name	Description	# unique insertions	Log_2 enrichment	Subcellular localization
λ	*malT*	transcriptional regulator MalT (NCBI)	135	6.57	Cytoplasmic
λ	*lamB*	maltoporin precursor (NCBI)	64	6.37	Outer Membrane
λ	*malI*	DNA-binding transcriptional repressor (NCBI)	11	2.63	Cytoplasmic
λ	*malK*	fused maltose transport subunit (NCBI)	24	1.30	Cytoplasmic
λ	*b1355*	orf, hypothetical protein (VIMSS)	5	1.18	
λ	*slyA*	transcriptional regulator for cryptic hemolysin (VIMSS)	3	1.18	Cytoplasmic
λ	*ycfP*	orf, hypothetical protein (VIMSS)	3	1.16	Cytoplasmic
λ	*manY*	mannose-specific enzyme IIC component of PTS (NCBI)	8	1.12	Integral Inner Membrane
λ	*rfaF*	ADP-heptose:LPS heptosyltransferase II (NCBI)	62	1.09	Cytoplasmic
λ	*manZ*	mannose-specific enzyme IID component of PTS (NCBI)	16	1.08	Integral Inner Membrane
λ	*azoR*	acyl carrier protein phosphodiesterase (NCBI)	4	0.97	Cytoplasmic
Bas14	*metQ*	DL-methionine transporter subunit (NCBI)	25	5.51	Inner Membrane Lipoprotein
Bas14	*secB*	export protein SecB (NCBI)	14	1.17	Cytoplasmic
Bas14	*yneL*	predicted transcriptional regulator (NCBI)	7	0.20	Cytoplasmic
Bas14	*yaiS*	orf, hypothetical protein (VIMSS)	28	0.05	Cytoplasmic
TMP(A105E)	*metQ*	DL-methionine transporter subunit (NCBI)	25	11.14	Inner Membrane Lipoprotein
TMP(A105E)	*secB*	export protein SecB (NCBI)	14	0.72	Cytoplasmic
TMP(A105E)	*yjgR*	predicted ATPase (NCBI)	61	0.28	Cytoplasmic
TMP(A105E)	*tufB*	protein chain elongation factor EF-Tu (NCBI)	7	0.27	Cytoplasmic

List of host genes required for infection of λ, Bas14, and Bas14 *TMP(A105E)*, as identified via Tn-Seq. The number of unique transposon insertions per gene in the original pool, prior to selection, is indicated. Log2 enrichment scores refer to the averaged enrichment scores of transposons within a gene of interest after selection with a specific phage. Only genes with enrichment scores >0.95 are shown for λ, and ≥0.2 for Bas14 and Bas14 TMP*(A105E)*. The predicted subcellular localizations of gene products, as determined by UniProt[54], are shown.

Supplementary Table 2

	*SNIPE-FLAG(W257R I308V N250*pBPA*)* + Bas14		*SNIPE-FLAG(N250*pBPA*)* + Bas14	
	Replicate 1	Replicate 2	Replicate 1	Replicate 2
gp18(TMP) spectral counts	2	5	0	0
gp8(MCP) spectral counts	0	26	0	0

Spectral counts of all phage proteins identified via mass spectrometry for Fig 4e and Extended Data Fig. 8b.

Supplementary Table 3

SNIPE homologs with 1-2 predicted TMs

Hits	*Protein ID*	*Domain name*
116	PF06785.14	UPF0242
66	6N2Y_b2	ATP synthase subunit b
32	8BH1_E	FtsB
21	3JC9_Oc	PilO
14	6S7T_E	Oligosaccharyltransferase
11	PF05103.16	DivIVA
10	PF06667.15	PspB
6	PF10828.11	DUF2570
4	cd06503	ATP synthase subunit b
4	4ZXQ_A	Phage P22 tail protein

SNIPE homologs with no predicted TMs

Hits	*Protein ID*	*Domain name*
44	6GAP_B	Reovirus tail protein
27	PF05103.16	DivIVA
17	PF10708.12	DUF2510
10	PF06785.14	UPF0242
6	PF18269.4	T3SS ATPase_C

Most frequent HMMER hits for the N-terminal regions of SNIPE homologs with 1-2 predicted TMs (left) or with no predicted TMs (right). Details of the hit count calculation are provided in the Methods section.

51. El Yaagoubi, A., Kohiyama, M. & Richarme, G. Localization of DnaK (chaperone 70) from Escherichia coli in an osmotic-shock-sensitive compartment of the cytoplasm. J. Bacteriol. 176, 7074–7078 (1994).
52. Pages, J. M., Bolla, J. M., Bernadac, A. & Fourel, D. Immunological approach of assembly and topology of OmpF, an outer membrane protein of Escherichia coli. Biochimie 72, 169–176 (1990).
53. Gilchrist, C. L. M. & Chooi, Y.-H. clinker & clustermap.js: automatic generation of gene cluster comparison figures. Bioinformatics 37, 2473–2475 (2021).
54. The UniProt Consortium et al. UniProt: the Universal Protein Knowledgebase in 2025. Nucleic Acids Res. 53, D609–D617 (2025).
