# Supplementary material for: A membrane-bound nuclease directly cleaves phage DNA during genome injection
Source: Nature. 2026 Feb 25;653(8115):861–9. doi: 10.1038/s41586-026-10207-1 (PMC13190303; doi:10.1038/s41586-026-10207-1)
Supplement: Supplementary file 2 — Reporting Summary [file 41586_2026_10207_MOESM2_ESM.pdf]

nature portfolio

Corresponding author(s): Michael T. Laub

Last updated by author(s): Jan 16, 2026

Reporting Summary

Nature Portfolio wishes to improve the reproducibility of the work that we publish. This form provides structure for consistency and transparency in reporting. For further information on Nature Portfolio policies, see our Editorial Policies and the Editorial Policy Checklist.

Statistics

For all statistical analyses, confirm that the following items are present in the figure legend, table legend, main text, or Methods section.

n/a	Confirmed	
☐	☒	The exact sample size (*n*) for each experimental group/condition, given as a discrete number and unit of measurement
☐	☒	A statement on whether measurements were taken from distinct samples or whether the same sample was measured repeatedly
☐	☒	The statistical test(s) used AND whether they are one- or two-sided<br>*Only common tests should be described solely by name; describe more complex techniques in the Methods section.*
☒	☐	A description of all covariates tested
☒	☐	A description of any assumptions or corrections, such as tests of normality and adjustment for multiple comparisons
☐	☒	A full description of the statistical parameters including central tendency (e.g. means) or other basic estimates (e.g. regression coefficient) AND variation (e.g. standard deviation) or associated estimates of uncertainty (e.g. confidence intervals)
☐	☒	For null hypothesis testing, the test statistic (e.g. *F*, *t*, *r*) with confidence intervals, effect sizes, degrees of freedom and *P* value noted<br>*Give P values as exact values whenever suitable.*
☒	☐	For Bayesian analysis, information on the choice of priors and Markov chain Monte Carlo settings
☒	☐	For hierarchical and complex designs, identification of the appropriate level for tests and full reporting of outcomes
☐	☒	Estimates of effect sizes (e.g. Cohen's *d*, Pearson's *r*), indicating how they were calculated

Our web collection on statistics for biologists contains articles on many of the points above.

Software and code

Policy information about availability of computer code

Data collection	Consurf 2016 web server, FoldSeek Release 10, HHsuite v3.3.0
Data analysis	MMseqs2 Release 14, UCSF ChimeraX v1.7, pandas v2.0.3, numpy v1.24.4, matplotlib v3.2.2, seaborn v0.10.1, Geneious version 2025.3, Fiji (ImageJ) version 2.1.0: watershed algorithm and coloc 2 plugin, AlphaFold version 3 web server

For manuscripts utilizing custom algorithms or software that are central to the research but not yet described in published literature, software must be made available to editors and reviewers. We strongly encourage code deposition in a community repository (e.g. GitHub). See the Nature Portfolio guidelines for submitting code & software for further information.

Data

Policy information about availability of data

All manuscripts must include a data availability statement. This statement should provide the following information, where applicable:

- Accession codes, unique identifiers, or web links for publicly available datasets
- A description of any restrictions on data availability
- For clinical datasets or third party data, please ensure that the statement adheres to our policy

Sequencing data is available in the Sequence Read Archive under BioProject PRJNA1231458. Summaries of spectral read counts and raw data for MS/MS of biotinylated or crosslinked proteins were deposited under MassIVE and can be accessed under accession MSV000097285. All other data are available in the manuscript or supplementary materials. Source data are provided for Figures 1-5 and Extended Data Figures 1, 3, 4, 6-10.

Research involving human participants, their data, or biological material

Policy information about studies with human participants or human data. See also policy information about sex, gender (identity/presentation), and sexual orientation and race, ethnicity and racism.

Reporting on sex and gender	N/A
Reporting on race, ethnicity, or other socially relevant groupings	N/A
Population characteristics	N/A
Recruitment	N/A
Ethics oversight	N/A

Note that full information on the approval of the study protocol must also be provided in the manuscript.

Field-specific reporting

Please select the one below that is the best fit for your research. If you are not sure, read the appropriate sections before making your selection.

☒ Life sciences ☐ Behavioural & social sciences ☐ Ecological, evolutionary & environmental sciences

For a reference copy of the document with all sections, see nature.com/documents/nr-reporting-summary-flat.pdf

Life sciences study design

All studies must disclose on these points even when the disclosure is negative.

Sample size	No formal sample size calculation was performed. Sample sizes were selected based on prior experience and expected effect sizes, aiming to detect biologically meaningful differences while demonstrating reproducibility. Each experiment was independently replicated 2–4 times, which we determined is sufficient given the consistently large effect sizes observed.
Data exclusions	No data were excluded.
Replication	All experiments, except those noted below, were independently repeated at least twice, and all attempts were successful. TurboID with mass spectrometry experiments were only performed once due to cost and significant overlap across the reported experiments. Tn-Seq was only performed once due to recovery of expected hits such as ManY and ManZ with phage λ.
Randomization	All experiments were performed in isogenic strains so there were no covariates to control for. No subjective choice of experimental and control groups was performed.
Blinding	Blinding was not considered necessary because all data were objective and quantitative, such as sequencing reads, mass spectrometry intensities, or discrete counts. Raw data are reported in full in the manuscript, and all experiments were independently replicated with consistent results, minimizing the potential for bias in data collection or analysis.

Reporting for specific materials, systems and methods

We require information from authors about some types of materials, experimental systems and methods used in many studies. Here, indicate whether each material, system or method listed is relevant to your study. If you are not sure if a list item applies to your research, read the appropriate section before selecting a response.

Materials & experimental systems

n/a	Involved in the study	
☐	☒	Antibodies
☒	☐	Eukaryotic cell lines
☒	☐	Palaeontology and archaeology
☒	☐	Animals and other organisms
☒	☐	Clinical data
☒	☐	Dual use research of concern
☒	☐	Plants

Methods

n/a	Involved in the study	
☒	☐	ChIP-seq
☒	☐	Flow cytometry
☒	☐	MRI-based neuroimaging

Antibodies

Antibodies used

GFP Monoclonal (3E6) Mouse mAb, Invitrogen, Cat#: A11120
E. coli DnaK (1-384 aa) Rabbit pAb, AssayPro, Cat#: 32857-05111
OmpC Rabbit pAb, Bioss Antibodies, Cat#: bs20213R
Goat anti-Mouse IgG (H+L) Secondary Antibody, HRP, Thermo Fisher, Cat #: 32430
Goat anti-Rabbit IgG (H+L) Secondary Antibody, HRP, Thermo Fisher, Cat #: 32460

Validation

Primary antibodies were validated both by the manufacturer and in our experiments. Manufacturer validation statements (from the supplier websites) are as follows:
anti-GFP: "Antibody specificity was demonstrated by detection of different targets fused to GFP tag in transiently transfected lysates tested."
anti DnaK: "Assay was performed using increasing concentrations of biotinylated recombinant E. coli DnaK protein"
anti-OmpC: "E. coli lysates probed with OmpC Polyclonal Antibody"
anti-Mouse: "Western blot analysis of HA Epitope Tag performed by various amounts of E. coli lysate containing a multi-epitope tagged protein"
anti-Rabbit: "Western blot analysis performed on membrane enriched extracts of K562 and PC-3."
In addition, we confirmed that each antibody produced a single band at the expected molecular weight in our samples and detected proteins in the appropriate cellular fraction (cytoplasmic or membrane), consistent with prior literature. Negative controls lacking the target protein produced no signal, supporting specificity.

Plants

Seed stocks

Report on the source of all seed stocks or other plant material used. If applicable, state the seed stock centre and catalogue number. If plant specimens were collected from the field, describe the collection location, date and sampling procedures.

Novel plant genotypes

Describe the methods by which all novel plant genotypes were produced. This includes those generated by transgenic approaches, gene editing, chemical/radiation-based mutagenesis and hybridization. For transgenic lines, describe the transformation method, the number of independent lines analyzed and the generation upon which experiments were performed. For gene-edited lines, describe the editor used, the endogenous sequence targeted for editing, the targeting guide RNA sequence (if applicable) and how the editor was applied.

Authentication

Describe any authentication procedures for each seed stock used or novel genotype generated. Describe any experiments used to assess the effect of a mutation and, where applicable, how potential secondary effects (e.g. second site T-DNA insertions, mosiacism, off-target gene editing) were examined.
